# Artificial Intelligence Remote Patient Monitoring for Predicting Overall Survival for Patients Undergoing Radical Cystectomy for Bladder Cancer: Exploratory Analysis of the Prospective Trial

**DOI:** 10.2196/68994

**Published:** 2026-05-20

**Authors:** Yansong Liu, Pramit Khetrapal, Ronnie Strafford, Adamos Hadjivasilou, Nikhil Vasdev, Philip Charlesworth, Muhamaad Shamim Khan, Ahmed Abdulaal, Zhaoyan Dong, James W F Catto, Yukun Zhou, John D Kelly, Ivana Drobnjak

**Affiliations:** 1 Centre for Artificial Intelligence, Department of Computer Science, University College London, 1st Floor, 90 High Holborn, London, WC1V 6LJ, United Kingdom, 44 07939274833; 2 Department of Targeted Intervention, Division of Surgery & Interventional Science, University College London, London, United Kingdom; 3 Hertfordshire and Bedfordshire Urological Cancer Centre, Lister Hospital, Stevenage and School of Life and Medical Sciences, University of Hertfordshire, Hatfield, United Kingdom; 4 Royal Marsden NHS Foundation Trust, London, United Kingdom; 5 Department of Urology, Guy's and St Thomas' NHS Foundation Trust, London, United Kingdom; 6 Division of Clinical Medicine, School of Medicine & Population Health, University of Sheffield, Sheffield, United Kingdom

**Keywords:** remote patient monitoring, artificial intelligence, wearable device, survival prediction, radical cystectomy, machine learning, mobile phone, AI RPM

## Abstract

**Background:**

Previous studies have highlighted the benefits of using artificial intelligence–powered remote patient monitoring (AI RPM) in detecting health changes across various disease cohorts. However, the use of AI RPM for identifying health deteriorations in patients following major surgical procedures remains underexplored.

**Objective:**

This exploratory analysis of a prospective trial aims to assess how AI RPM can enhance the predictive performance of 35-month post–radical cystectomy (RC) mortality risk. Our approach highlights the importance of RPM features in improving prediction accuracy and provides interpretable model outputs to enhance clinical understanding and transparency.

**Methods:**

We used patient data from a multicenter RC trial conducted in the United Kingdom for model training and validation. Two gradient-boosted machine learning models were developed: one using only clinical-pathological (CP) features and another incorporating both CP and remote patient monitoring (RPM) features (CP+RPM). RPM features are measured by wrist-worn pedometers and surveys. The predictive accuracy of the CP+RPM model was compared with both the CP model and a clinically used nomogram, both of which relied solely on traditional clinical features. We used 200 bootstrap iterations, with 70% of the data used for training and 30% for testing. Shapley Additive Explanations were applied to interpret model results and provide insights into the relative importance of features, improving transparency and understanding of the predictions.

**Results:**

A total of 252 patients (33 deaths) from 9 UK centers were included in the analysis. We examined 108 RPM features and 24 CP features for model training. In correlation analysis, only 9 CP features showed coefficients larger than 0.1, compared with 36 RPM features with stronger correlations. The CP+RPM model achieved an area under the receiver operating characteristic curve of 0.77, reflecting a 9% and 10% absolute (13% and 15% relative) improvement over the CP and nomogram models, respectively. Similarly, it outperformed in terms of the area under the precision-recall curve, with a score of 0.44, marking a 6% and 17% absolute (16% and 63% relative) increase compared with the CP and nomogram model. Shapley Additive Explanations analysis revealed that the most significant contributors to mortality prediction were mobility-related RPM features, such as the 30-second chair-to-stand test results and daily step count variance, which reflected the general activity levels of individuals.

**Conclusions:**

Our study demonstrates that RPM features significantly enhance long-term survival prediction for post-RC patients, offering a valuable addition to traditional clinical data. The integration of AI with RPM enables more individualized and dynamic tracking of recovery, improving prediction accuracy and fostering a patient-centered care model that has the potential to be applied across a broader range of surgeries and conditions.

## Introduction

Surgery plays a key role in cancer treatment, with 45% of cancer cases treated surgically [1] and more than 8 million cancer surgeries performed globally each year [2]. Monitoring and understanding the survival probability following a surgery are crucial for clinical decisions, planning future treatment pathways, and managing patient care [3]. A commonly used statistical tool for predicting overall survival is the nomogram, which calculates survival probability based on patient-specific characteristics [4]. Nomograms are considered the gold standard in clinical prediction models and are widely used in clinical practice [5]. However, major disadvantages such as suboptimal performance and lack of individualization limit nomograms from being reliably used for personalized medicine.

Recently, artificial intelligence (AI) and data science have gained popularity in the medical field [6]. AI has enhanced model performance and individualized predictions across a variety of applications. For example, Sun et al [7] explored the use of AI language models for extracting clinical records, improving the accuracy of 5-year survival predictions in bladder cancer surgery patients. Similarly, Lou et al [8] developed an AI-based 10-year survival prediction model for patients receiving breast cancer surgery, and Osman et al [9] showed that AI-based models perform better than some traditional models when predicting 5-year survival for patients with colorectal cancer. Although these models have shown promising results, most of them are limited to in-hospital data only, limiting their scope. Expanding AI models to incorporate at-home data could significantly enhance individual health outcomes and public health by enabling remote, continuous, and long-term monitoring.

Remote patient monitoring (RPM) is an emerging technology that enables collecting data from patients outside the hospital setting. With advances in wearables, sensors, high-speed internet, and smartphones, RPM has emerged as a valuable tool for improving predictive accuracy and tailoring treatments to individual needs [10]. RPM data include metrics such as steps, heart rate, blood glucose level, sleep quality, mood, and quality of life [11,12]. These data are continuously transmitted between patients and health care providers. However, the volume and complexity of RPM data can be overwhelming, often making it difficult for health care providers to interpret and act upon the data in real time. This can lead to inconsistencies in care, where critical insights might be missed or delayed.

By integrating AI with RPM (AI RPM), clinicians can gain a powerful tool that not only processes large datasets but also analyzes and synthesizes the information to find patterns and trends and provide actionable insights, enabling more precise and timely interventions [13,14]. This approach has the potential to significantly improve health outcomes by offering more personalized care, reducing hospital admissions, and allowing for earlier detection of health issues. Additionally, AI RPM can optimize resource allocation and reduce health care costs by targeting interventions where they are most needed, ultimately supporting a more efficient and patient-centered health care model.

In this study, we developed a novel AI RPM model for patients with bladder cancer who underwent cystectomy, a procedure involving the removal of the bladder and prostate. Patients undergoing radical cystectomy (RC) often experience a prolonged and heterogeneous recovery period, with substantial variation in physiological stability, functional status, and perceived well-being after discharge [15,16]. In addition to the physiological burden of major surgery, many patients manage complex urinary diversions and have multiple comorbidities that increase vulnerability to postoperative complications, making close postdischarge monitoring clinically important but difficult to achieve through conventional follow-up alone.

The data used in this exploratory study were extracted from the iROC (intracorporeal robot-assisted radical cystectomy vs open radical cystectomy) trial [17], a prospective multicenter randomized controlled trial involving 317 patients who underwent RC for bladder cancer across the United Kingdom from March 2017 to January 2020. The AI RPM model uses biometric data, quality-of-life measures, and clinical pathological data collected within the first 12 weeks postsurgery to predict survival outcomes. The model’s predictions and internal feature weights were explained using Shapley Additive Explanations (SHAP) for transparency and clinical relevance. Our study demonstrates the potential of AI RPM to be applied to various cancer surgeries, supporting its integration into clinical decision-making to enhance personalized care.

## Methods

### Data Source and Study Design

The data used in this exploratory study were extracted from a prospective, multicenter randomized controlled trial—iROC [17]. The iROC trial involved 317 patients with clinical N1 or N0 stage bladder cancer who underwent RC across the United Kingdom from March 2017 to January 2020. The inclusion and exclusion criteria for patients are detailed in Multimedia Appendix 1.

The primary aim of the iROC clinical trial was to compare patient outcomes between robotic and open cystectomy surgery. This study builds upon that by testing new hypotheses: “Does the AI RPM model improve the prediction of long-term survival in cystectomy patients?” Both robotic and open surgery patients were included in the analysis, and the surgical approach was captured as one of the features.

The model used data collected within the first 12 weeks following surgery to predict the risk of all-cause mortality within 35 months. The 12-week window was selected based on clinical considerations, as the early post-RC period is recognized as the most vulnerable phase, during which the majority of complications and hospital readmissions occur [18,19]. While a longer RPM observation window may potentially provide additional information, extending the monitoring period is likely to yield diminishing gains in predictive performance while substantially increasing patient burden. Therefore, this time window represents a pragmatic balance between information content and feasibility.

A combination of RPM-derived features and traditional clinical and pathological variables was extracted from this 12-week period and used as model inputs. The model outputs a probability of mortality within 35 months, taking values between 0 and 1, where higher values indicate greater mortality risk. For descriptive and evaluation purposes only, a probability threshold of 0.5 was applied to dichotomize patients into high- and low-risk groups; however, all model predictions were generated as probabilities rather than binary outcomes.

To evaluate the added value of RPM data, the performance of the AI-based RPM model was compared with baseline models that used only traditional clinical and pathological features. This comparison quantified the incremental predictive benefit attributable to the integration of RPM-derived information.

### Ethical Considerations

The iROC trial received ethical approval from the Newcastle and North Tyneside Research Ethics Committee (reference 16/NE/0418), was sponsored by University College London, and was prospectively registered before commencement. The trial was overseen by independent trial steering and data-monitoring committees. This secondary analysis was covered under the same ethical approval. Written informed consent was obtained from all participants before enrollment and onboarding to the remote patient-monitoring program. Participants were informed of the study objectives, procedures, potential risks, and their right to withdraw at any time without affecting their clinical care. Participant privacy and confidentiality were protected throughout the study. All data were deidentified or pseudonymized prior to analysis, stored securely on password-protected systems with access restricted to authorized study personnel, and handled in accordance with applicable data protection regulations. No financial compensation was provided to participants for participation in this study.

### Data Extraction and Preprocessing

**Figure 1.**
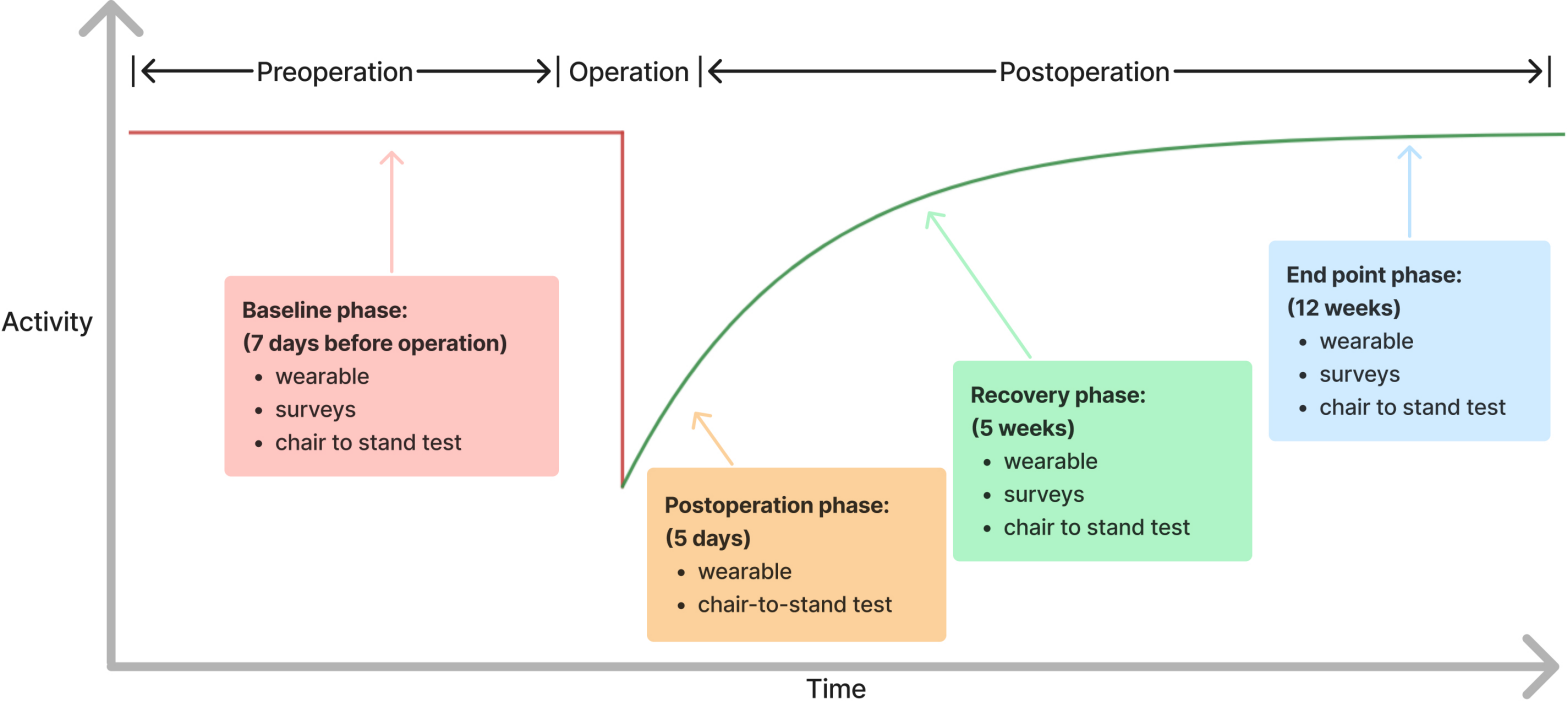
Expected activity level in preoperative and postoperative periods. Patient activity level drops after receiving the surgery and then gradually returns to preoperative baseline as the patient recovers. Different types of data (outlined in the box) were collected in a series of monitoring phases (ie, baseline, postoperation, recovery, and end point).

The original RPM data from the iROC study was collected over 6 phases to capture trends in preoperative and postoperative activity levels [20,21]. To enable early detection of mortality risk, only the first 4 phases (Figure 1) were considered in this study. The first phase (baseline phase) started on the day of enrollment. After surgery, patients received 3 more postoperative monitoring phases (postoperative phase, recovery phase, and end point phase) to capture the postoperative recovery level. In all 4 phases, daily step count and 30-second chair-to-stand (CTS) test were collected. CTS test is a common method of assessing lower body strength in older adults, which measures the number of sit-to-stand repetitions the participant can perform within 30 seconds [22]. MisFit Ray [23] pedometers were issued to patients in the clinic after face-to-face follow-up. After the 7-day monitoring period, patients mailed the pedometer back for data extraction. A photograph of the wearable devices kit is displayed in Multimedia Appendix 2.

In addition to the activity data, surveys were administered at the baseline, postoperative, and recovery phases. Three surveys were included. The first one was the European Quality of Life group (EQ-5D-5L) [24], consisting of 6 questions on quality of life. The first 5 questions focused on mobility, self-care, usual activity, and pain or discomfort and anxiety or depression. Each question is scored 1‐5, with 5 being the best. The last question is a European Quality of Life visual analogue scale that captures the respondent’s overall assessment of their health on a scale from 0 (worst health imaginable) to 100 (best health imaginable). The second one is European Organisation for Research and Treatment of Cancer (EORTC) QLQ-C30 [25]—a 30-question quality-of-life instrument developed for self-evaluation of patients with generic cancer. The questionnaire is grouped into (1) Functional evaluation (physical, role, emotional, cognitive, social, etc) consisting of 15 questions with scores 1 (worst) to 4 (best), (2) Symptom/Problems evaluation (fatigue, nausea or vomiting, pain, dyspnea, insomnia, appetite loss, constipation, diarrhea, and financial difficulty) consisting of 13 questions with scores 1 (best) to 4 (worst), and (3) Global Health status consisting of 2 questions with scores 1 (worst) to 7 (best). Finally, the third questionnaire is the EORTC QLQ-BLM30, a 30-item quality-of-life survey targeted for patients with bladder and prostate cancer that can be attached to QLQ-C30 to enrich the measurement comprehensiveness. QLQ-BLM30 assesses urinary problems, urostomy problems, abdominal problems, future perspective, overall body image, and catheter use problems. Each question scored from 1 to 4, with 4 being the best. Further details of all 3 questionnaires are provided in Multimedia Appendix 3.

Patient data were extracted from the date of patient consent through to the end of the end point phase. The following data categories were collected:

1. Demographics data: sex, age, height, weight, eastern cooperative oncology group (ECOG) performance status score [26].
2. Pathological data: T stage, tumor grade, is tumor a flat carcinoma in situ, surgery margin clear.
3. Clinical data: clinical N stage, cancer type, pre-operative chemotherapy date, pre-operative immunotherapy date.
4. Surgical data: surgery type (open or robotic surgery), surgery duration, surgery blood loss in milliliters, other body parts injured by surgery, number of nodes resected, number of positive nodes among resected nodes.
5. Inpatient stay data: length of stay, nights in standard ward, nights in intensive care unit, nights in high dependency unit, number of theater visits, days taken to return to regular diet.
6. RPM data: daily step count, CTS performance, EQ-5D-5L, QLQ-C30, and QLQ-BLM30 responses.
7. Mortality data: death dates of deceased patients (gathered during follow-ups with final follow-up on September 23, 2021).

The first 5 data categories have been traditionally used in nomograms and are categorized as “clinical-pathological (CP)” data. The sixth category, which includes RPM results, is referred to as “RPM” data. Although the surveys and CTS test were conducted face-to-face, they can also be completed remotely without professional assistance and were thus classified as RPM data in this analysis.

The following preprocessing steps were performed on the extracted data: (1) height and weight were converted to BMI, (2) the cancer type was encoded using one-hot encoding, (3) preoperative chemotherapy date and preoperative immunotherapy date were converted to days relative to the day of surgery, and (4) the original “other body parts injured by surgery” variable was aggregated with “did the surgery injure any other body part.”

Individual survey answers were converted to aggregated survey scores using officially validated, internationally recognized methods. The first survey, EQ-5D-5L, was processed using the England weighting system [27]. The first 5 responses were mapped to corresponding component scores and then summed to generate an overall quality-of-life score, ranging from −0.285 (worse than death) to 1.0 (best imaginable quality of life). The second survey, QLQ-C30, was scored using the EORTC QLQ-C30 integrated system [25]. For each of the 3 subdomains—Global Health Status, Functional Scales, and Symptom Scales—individual scale scores were calculated by averaging the relevant question responses (as in Multimedia Appendix 3), followed by a linear transformation to map the scores onto a 0‐100 range. For Functional Scales and Global Health Status, a score of 0 indicated the worst outcome and 100 the best, while for Symptom Scales, 0 represented the best condition and 100 the worst. The third survey, QLQ-BLM30, was scored similarly to the QLQ-C30 Symptom Scales.

Finally, the following filtering steps were performed to remove outliers and patients who are not suitable for analysis: (1) participants who unfortunately passed away before completing all 4 phases were excluded; (2) due to the impact of the COVID-19 pandemic, the collection of remote activity data was halted early for safety concerns; those affected by the early termination were excluded; and (3) any phase with 2 days or fewer daily step count records during a monitoring phase (7 days in each phase) was marked as entirely missing due to low reliability. Features derived from daily step counts of this patient in this phase were also marked as missing.

### Feature Engineering

Feature engineering is crucial in machine learning as it enables models to capture important patterns in the data that may not be immediately obvious, leading to more effective and accurate predictions. By transforming raw data into more informative features, the model can better identify relationships that standard data might overlook. The following feature engineering steps were performed: (1) *Aggregation of daily step count*: We aggregated daily step counts by monitoring phases. For each phase, we calculated the mean, variance, minimum, and maximum values. Note that these features were used in place of raw daily step count. This method not only streamlined the input data but also mitigated the issue of missing data in the original daily counts. (2) *Patient compliance*: To further enhance our model’s interpretability, we took into account the missing ratio of daily step counts for each phase, as well as the overall missing ratio across all 4 phases. This allowed us to assess patient compliance in wearing the monitoring device. (3) *Recovery trends*: to enhance our analysis of recovery patterns, we calculated the ratio of mean step counts in each phase relative to the previous phases. The same process was applied to the CTS test results.

All features were standardized using the minimum-maximum scaler to ensure comparable feature magnitude. Univariate Kendall tau correlation analyses were then conducted to assess the association between each feature and patient survival. Several variables exhibited nonnormal distributions (eg, step count showed a zero-inflated distribution, and the CTS test results were non–normally distributed). Kendall tau was therefore chosen as a nonparametric rank-based measure that is robust to nonnormality, tied values, and small sample sizes. Pairwise correlations between features were also evaluated. Features with a correlation coefficient exceeding 0.6 with another feature were flagged as collinear. In each of the collinear feature pairs, the feature with a weaker correlation to patient survival was discarded. The final features were selected based on their correlation ranking with survival outcomes.

### Statistical Analysis of Features

The raw features were firstly explored with statistical approaches. Continuous features were expressed as mean with IQR, and survivor vs nonsurvivor groups were compared using the Mann-Whitney *U* test. Categorical features were expressed as numbers and percentages relative to the whole group and compared using chi-square tests.

### Model Development and Validation

The primary objective of this study was to quantify the incremental predictive value of RPM features through predicting mortality risk at 35 months after RC. A gradient boosting machine (GBM) was selected because it can model complex, nonlinear interactions while remaining well suited to small to moderate sample sizes [28]. The dataset comprises 252 patients with 33 mortalities and a limited number of engineered summary features derived from discrete postoperative time points rather than high-resolution longitudinal signals; under these conditions, more complex models such as deep learning are prone to overfitting and offer limited practical benefit. In addition, the data exhibit missing-not-at-random patterns related to recovery and device adherence, making modeling approaches that rely on explicit imputation, including standard Cox proportional hazards models, less appropriate for this study. GBM can natively incorporate missingness as informative splits during training. Finally, when combined with SHAP, GBM provides excellent interpretability, enabling transparent examination of feature contributions without sacrificing predictive performance. This aligns with the study’s primary aim of quantifying the incremental predictive value of RPM features, rather than optimizing peak performance across multiple advanced model families.

The primary model, referred to as the CP+RPM model, was developed using a combination of CP data and RPM data, enabling a more comprehensive approach. The model outputs diagnostic binary classification results, predicting either high or low risk of death within the observation period, along with corresponding probabilities. To quantify the performance improvement from adding RPM features, we developed a baseline model using only CP data, referred to as the CP model. Both CP+RPM and CP models were trained with a 7.69:1 positive-to-negative class weight ratio to address class imbalance. This weight ratio was chosen to reflect the 13% positive class ratio in the dataset, ensuring that the models are not biased toward the majority negative class. Additionally, a nomogram-based model commonly used by clinicians for predicting survival in patients with bladder cancer was included in the evaluation for comparison purposes [29]. Limited manual optimization of hyperparameters was performed. Given the small sample size, we avoided automated or exhaustive hyperparameter tuning to reduce the risk of overfitting. Instead, we followed typical hyperparameter settings commonly reported in the literature. The same settings were used for all the models (where relevant). Hyperparameter configurations are provided in Multimedia Appendix 4.

**Figure 2.**
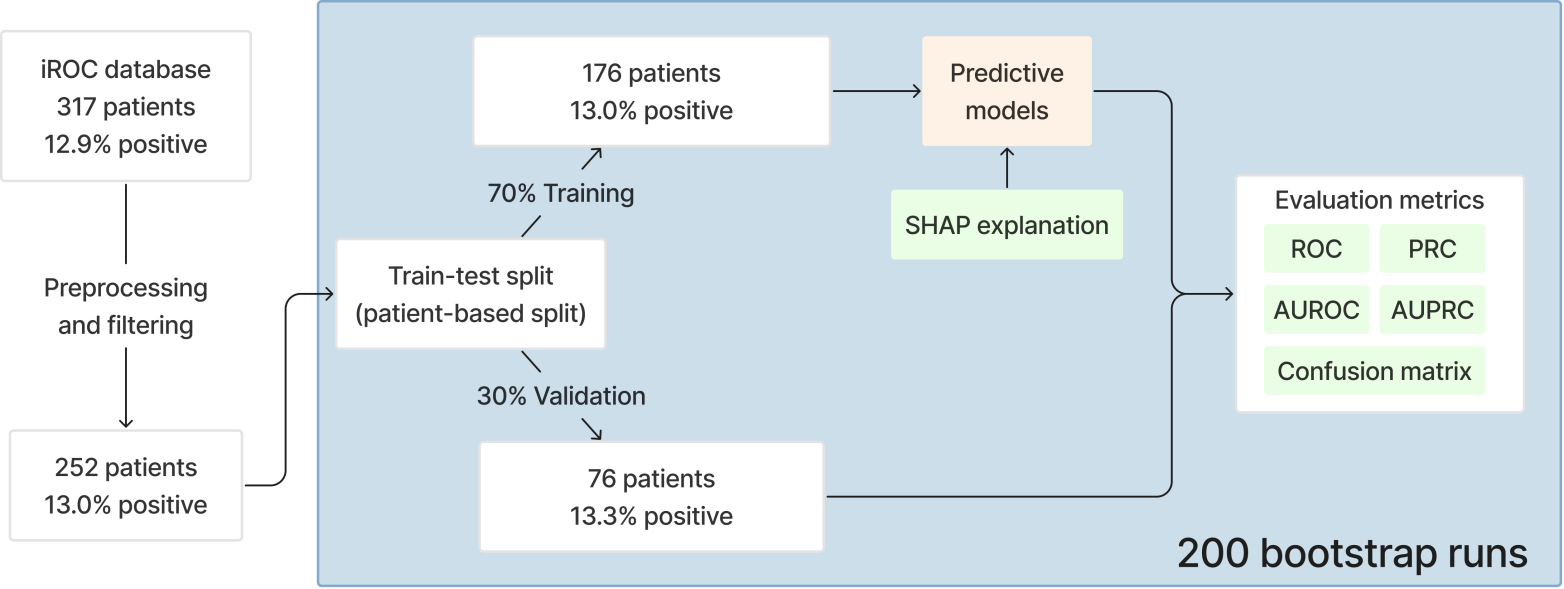
Data processing and analysis workflow. AUPRC: area under the precision-recall curve; AUROC: area under receiver operating characteristic curve; iROC: intracorporeal robot-assisted radical cystectomy vs open radical cystectomy; PRC: precision-recall curve; ROC: receiver operating characteristic curve; SHAP: Shapley Additive Explanations.

The patient data were randomly split into 2 subsets: 70% for model training (development set) and 30% for testing (validation set), as seen in Figure 2. The selection of patients into the 2 subsets was random while satisfying the percentage of positive cases in each subset to match the overall one (approximately 13%). The discriminative performance of each model for predicting mortality was evaluated using receiver operating characteristic (ROC) curves, with the area under the ROC curve (AUROC) used as a key metric to measure predictive accuracy. Precision-recall curves (PRCs), alongside the area under the precision-recall curve (AUPRC), were also reported to assess the model’s performance in handling imbalance classes, which is common in medical datasets. Confusion matrices were used to analyze the true positive, true negative, false positive, and false negative for each model. We calculated 95% CIs for these metrics using bootstrapping with 200 stratified bootstrap replicates [30] to ensure the robustness of the results. This approach helped quantify the uncertainty around the feature importance scores, providing a clearer picture of their reliability.

To enhance the interpretability of the trained models, SHAP was used to quantify the contribution of individual features to the model’s predictions [31]. SHAP is a post hoc explainability framework grounded in cooperative game theory, in which each feature is assigned an importance value (SHAP value) representing its contribution to the deviation of a specific prediction from the model’s baseline output. Positive SHAP values indicate that a feature increases the predicted risk of mortality, whereas negative values indicate a protective effect.

SHAP provides both global and local interpretability: global explanations provide an overview of feature importance across the cohort, while local explanations characterize how specific feature values influence predictions for individual patients. This approach is model-agnostic and is particularly suitable for complex machine learning models, allowing clinically meaningful interpretation without assuming linear relationships between features and outcomes.

In the context of our model, SHAP analysis explains how important each RPM-derived mobility metric or clinical characteristic is and to what degree they influenced mortality risk predictions. SHAP allows for detailed, case-specific explanations, highlighting the features that most influenced each patient’s risk score. This level of interpretability is essential for building clinician trust in AI models and making informed clinical decisions. Additionally, SHAP was applied at the individual prediction level to provide personalized interpretations for each patient, offering transparency in the model’s decision-making process.

### Case Analysis

To illustrate how the proposed CP+RPM model integrates various features to generate individualized mortality risk predictions, we performed a post hoc case analysis on representative positive and negative examples. These cases were selected from an independent test set and were not used during model training or hyperparameter tuning.

Predictions are expressed on the log-odds scale, with the model’s expected value serving as the baseline. Features with positive SHAP values (represented in red in the plot) increase the predicted mortality risk, whereas features with negative SHAP values (represented in blue in the plot) decrease the risk. The sum of all feature-specific SHAP values and the expected value produce a log-odds risk score. The score can be subsequently transformed into a predicted mortality probability via a separate logistic function.

## Results

### Patient Description

This study followed the “Guidelines for Developing and Reporting Machine Learning Predictive Models in Biomedical Research” [32]. A total of 317 patients from the iROC database were included in the initial extraction. Following preprocessing steps, 65 patients were excluded, leaving 252 patients for further analysis. The median observation period for these 252 patients was 35.0 months. During the observation period, 33 (13%) patients passed away, while 219 (87%) patients survived until the end of the study.

Tables1  2 provide a detailed comparison of the characteristics of survivors and nonsurvivors across different phases of monitoring. We see that the survivor group has a higher daily activity level in the recovery phase (*P*=.02). Survivors also performed better in the CTS tests in baseline, recovery, and end point phases (*P*=.044, *P*=.02, and *P*<.001, respectively). Moreover, survivors are better emotionally and cognitively in the end point phase (*P*=.04 and *P*=.043, respectively) and have lighter constipation and abdominal problems in the baseline phase (*P*<.001 and *P=*.009, respectively). Lower age, fewer positive nodes, lower length of stay, fewer days taken to return to a regular diet, and lower pathological T stage are also characteristics shown to be more prominent in the survival group.

**Table 1.** Characteristics of clinical features from patients following radical cystectomy at different phases of the follow-up. Both survival group and nonsurvival group characteristics are shown^a^.

Features	Survival group	Nonsurvival group	*P* value
Demographics			
Age (years), mean	68	73	<.001^b^
BMI (kg/m)^2^, mean	28	27	.56
Men, n	173	25	.80
Women, n	46	8	.80
ECOG^c^ (0 best, 4 worst), mean	0.32	0.45	.07
Cancer type, n			
Urothelial carcinoma	206	28	.12
Squamous carcinoma	24	7	.13
Adenocarcinoma	7	0	.63
Other	23	3	>.99
Surgical			
Robotic surgery, n	102	14	.80
Injury to other body parts, n	8	1	>.99
Margin clear, n	206	28	.12
Number of nodes resected, mean	16	15	.65
Number of positive nodes, mean	1	2	.006^d^
Inpatient stay, mean			
Length of stay	10	13.6	.04^e^
Number of theater visits	1.1	1	.28
Days taken to return to regular diet	6	8.4	.041^e^
Pathological			
T stage, n			
T0/Ta/Tis/Tx	84	5	.02^e^
T1	37	5	>.99
T2	48	5	.51
T3	40	13	.01^e^
T4	10	5	.045^e^
Other			
Flat carcinoma in situ, n	83	9	.41
Tumor grade, mean	2.9	3	.57

a Additional variables, including surgical and inpatient-related characteristics, as well as distribution measures (eg, IQRs and percentages), are provided in Multimedia Appendix 5.

b *P*<.001.

c ECOG: Eastern Cooperative Oncology Group performance status.

d *P*<.01.

e *P*<.05.

**Table 2.** Characteristics of remotely collected features from patients following radical cystectomy at different phases of the follow-up. Both survival group and nonsurvival group characteristics are shown^a^.

	Phase of collection											
	Baseline			Postoperation			Recovery			End point		
Features	Survival group	Nonsurvival group	*P* value	Survival group	Nonsurvival group	*P* value	Survival group	Nonsurvival group	*P* value	Survival group	Nonsurvival group	*P* value
Activity, mean (IQR)												
Daily step count x 0.001	6.6	6.4	.87	2	1.5	.11	4.8	3.4	.018^b^	6.2	5.2	.14
Chair-to-stand test in 30 seconds	13.7	12.3	.044^b^	4.1	3.2	.57	10.2	8.3	.024^b^	12.7	9.2	<.001^c^
Quality of life, score mean (IQR)												
** **EQ-5D-5L												
Quality of Life (−0.285 worst, 1.0 best)	0.9	0.91	.73	N/A^d^	N/A	N/A	0.81	0.77	.12	0.87	0.87	.67
EQ VAS^e^ (0 worst, 100 best)	80	82	.97	N/A	N/A	N/A	71	62	.054	80	72	.06
QLQ-C30^f^ functioning (0 worst, 100 best)												
Global health	22	23	.64	N/A	N/A	N/A	36	45	.089	25	31	.24
Physical functioning	91	92	.90	N/A	N/A	N/A	71	67	.43	83	78	.26
Role functioning	86	88	.55	N/A	N/A	N/A	54	53	.85	80	72	.21
Emotional functioning	81	87	.24	N/A	N/A	N/A	79	73	.24	83	76	.04^b^
Cognitive functioning	89	93	.26	N/A	N/A	N/A	85	80	.23	90	81	.043^b^
Social functioning	83	91	.12	N/A	N/A	N/A	63	63	.90	78	79	.72
QLQ-C30 symptoms (0 best, 100 worst)												
Fatigue	20	16	.48	N/A	N/A	N/A	42	47	.47	26	27	.76
Nausea and vomiting	4	3	.77	N/A	N/A	N/A	8	11	.10	4	5	.36
Pain	13	15	.56	N/A	N/A	N/A	26	33	.32	15	16	.59
Dyspnea	9	12	.59	N/A	N/A	N/A	19	28	.13	14	21	.12
Insomnia	27	23	.53	N/A	N/A	N/A	32	26	.25	22	31	.20
Appetite loss	9	11	.09	N/A	N/A	N/A	26	39	.11	1	24	.03
Constipation	12	28	<.001^c^	N/A	N/A	N/A	36	49	.091	22	25	.55
Diarrhea	4	2	.52	N/A	N/A	N/A	11	5	.16	8	11	.64
Financial difficulty	13	8	.42	N/A	N/A	N/A	18	8	.13	14	8	.49
QLQ-BLM30^g^ (0 best, 100 worst)												
Urinary problems	23	28	.12	N/A	N/A	N/A	26	11	.092	23	0	.18
Urostomy problems	N/A	N/A	N/A	N/A	N/A	N/A	12	14	.28	11	10	.35
Future perspective	31	25	.21	N/A	N/A	N/A	28	31	.77	22	27	.22
Abdominal problems	11	19	.009^h^	N/A	N/A	N/A	21	27	.10	16	17	.68
Body image	11	9	.78	N/A	N/A	N/A	21	14	.30	19	12	.08
Catheter use problem	N/A	N/A	N/A	N/A	N/A	N/A	2	0	.21	2	2	.53

a Distribution measures (eg, interquartile ranges and percentages) are provided in Multimedia Appendix 5..

b *P*<.05.

c *P*<.001.

d N/A: not applicable.

e EQ VAS: European Quality of Life visual analogue scale.

f QLQ-C30: European Organisation for Research and Treatment of Cancer QLQ-C30.

g QLQ-BLM30: European Organisation for Research and Treatment of Cancer QLQ-BLM30.

h *P*<.01.

Table 3 lists all the features that we engineered from the original variables. Nonsurvivors had less maximum daily step count in recovery and end point phases (*P=*.005 and *P*=.003, respectively). They also had smaller daily step count deviation over a monitoring period in recovery and end point phases (*P*=.005 and *P*=.004, respectively). Nonsurvivors generally recovered slower than survivors after receiving the cystectomy, measured by the result of the 30 seconds CTS test. Survivors, on average, recovered to the presurgery level or 1.7 times better than recovery phase by the end point phase. The nonsurvivors, however, recovered to only 70% of their presurgery baseline and performed worse than their recovery phase in their end point phase. This difference between 2 groups is statistically significant in end point phase relative to baseline phase (*P*=.007) and relative to recovery phase (*P*=.03).

**Table 3.** Characteristics of engineered remote patient monitoring features at different phases of the monitoring period. Both survival group and nonsurvival group characteristics are shown^a^.

	Phase of collection											
	Baseline			Postoperation			Recovery			End point		
	Survival group	Nonsurvival group	*P* value	Survival group	Nonsurvival group	*P* value	Survival group	Nonsurvival group	*P* value	Survival group	Nonsurvival group	*P* value
Daily step count × 0.001, mean												
Mean daily step count	6.6	6.4	.87	2.0	1.5	.11	4.8	3.4	.02	6.2	5.2	.14
Minimum daily step count	3.7	3.9	.73	1.0	0.8	.23	2.7	2.0	.052	3.4	3.0	.35
Maximum daily step count	9.8	9.4	.92	3.0	2.3	.08	7.4	4.9	.005^b^	9.1	6.8	.003^b^
SD	2.0	1.9	.64	0.7	0.5	.14	1.6	1.0	.005^b^	1.9	1.3	.004^b^
Recovery speed ratio, mean												
Mean daily step count												
To baseline	N/A^c^	N/A	N/A	0.4	0.4	.12	0.8	0.6	.13	1.0	0.2	.17
To postoperation	N/A	N/A	N/A	N/A	N/A	N/A	3.4	3.4	.55	4.6	4.5	.77
To recovery	N/A	N/A	N/A	N/A	N/A	N/A	N/A	N/A	N/A	1.6	2.2	.15
Chair-to-stand test in 30 seconds												
To baseline	N/A	N/A	N/A	0.4	0.2	.27	0.8	0.7	.31	1.0	0.7	.007^b^
To postoperation	N/A	N/A	N/A	N/A	N/A	N/A	5.3	4.5	.60	6.5	5.0	.39
To recovery	N/A	N/A	N/A	N/A	N/A	N/A	N/A	N/A	N/A	1.7	0.9	.03^d^
Compliance (%) of wearing, mean												
In each phase	69	74 (71‐100)	.60	73	65	.44	73	57	.07	63	57.0	.43

a Distribution measures (eg, IQRs and percentages) are provided in Multimedia Appendix 5. Mean overall compliance of wearing the device (through all 4 phases): survival group=70%; nonsurvival group=63% (*P*=.15).

b *P*<.01.

c N/A: not applicable.

d *P*<.05.

Survivors were also more compliant in wearing the wearable device. The compliance difference between groups is close to statistically significant in the recovery phase (*P*=.07). In that phase, survivors on average wore 73% (5.11 days) in the 7 days monitoring period, while nonsurvivors wore only 57% (3.99 days). Overall, survivors wore the wearable 70% of all the days, while nonsurvivors wore only 63% of that.

### Feature Ranking and Selection

**Figure 3.**
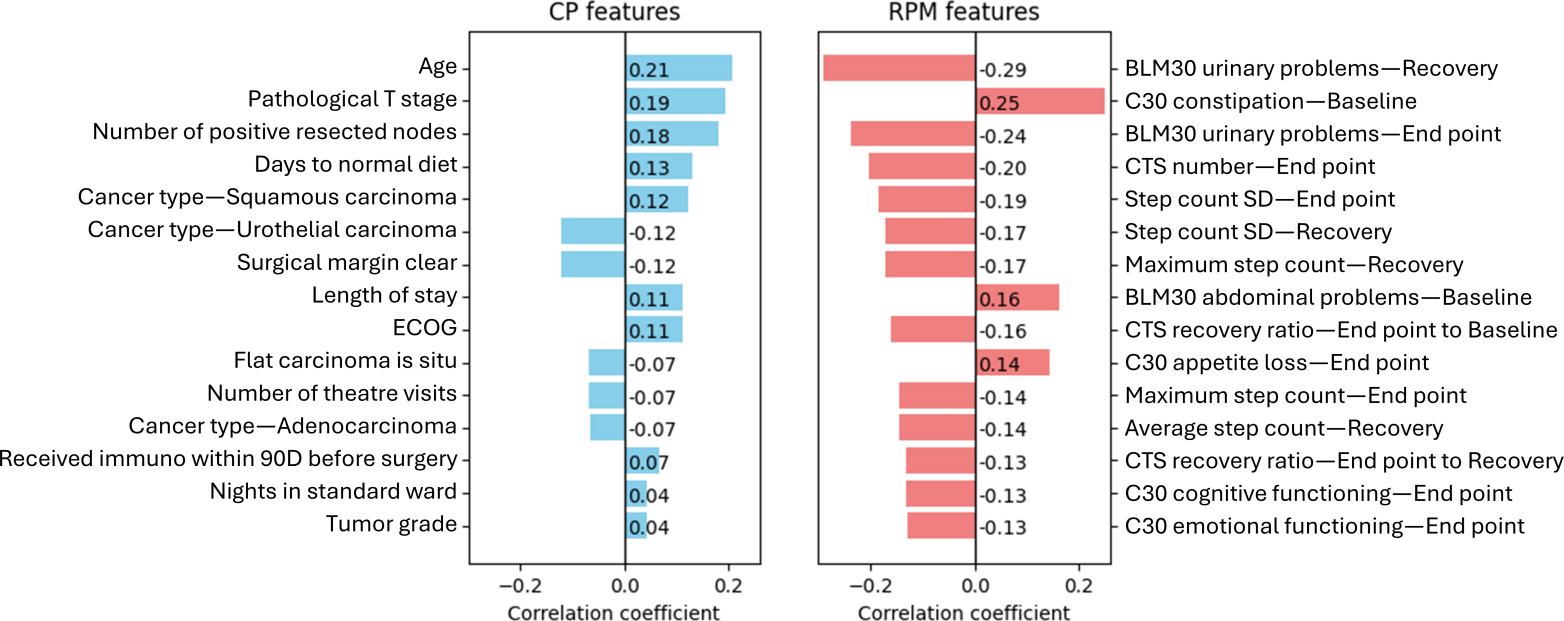
Top 15 features of each group, ranked by Kendall tau correlation coefficients. On the left (blue), are the CP features. On the right (red), are the RPM features. BLM30: European Organisation for Research and Treatment of Cancer QLQ-BLM30; C30: European Organisation for Research and Treatment of Cancer QLQ-C30; CP: clinical-pathological; CTS: chair-to-stand test; ECOG: Eastern Cooperative Oncology Group performance status; RPM: remote patient monitoring.

Figure 3 shows the ranking and correlation coefficient between the features and survival. It highlights the top 15 features with the strongest coefficient (the full correlation map is shown in Multimedia Appendix 6). Positive correlation coefficients means that as the feature value increases, the likelihood of death increases, that is, the feature is positively associated with mortality. A negative correlation coefficient means that as the feature’s value increases, the likelihood of death decreases (negative association with mortality). A larger absolute value of the correlation coefficient indicates a stronger correlation to survival. Note that the majority of RPM features (top 15) have absolute correlation coefficients larger than 0.1, whereas only 9 of the top 15 CP features exceed this threshold, implying at the minimum a measurable (although weak) relationship between the feature and survival.

In our 3 models we have used the highest scoring features listed in Figure 3. CP model used top 10 CP features (from the strongest to the weakest): pathological T stage, number of positive resected nodes, days taken to return to normal diet, cancer type—squamous carcinoma, cancer type—urothelial carcinoma, surgical margin clear, length of stay, ECOG, flat carcinoma in situ, and number of theater visits. CP+RPM model used top 10 CP+RPM features (from the strongest to the weakest): recovery phase QLQ-BLM30 urinary problems score, baseline phase QLQ-C30 constipation problems score, end point phase QLQ-BLM30 urinary problems score, end point phase CTS test result, pathological T stage, SD of end point phase daily step counts, number of positive resected nodes, SD of recovery phase daily step counts, baseline phase QLQ-BLM30 abdominal problems, and recovery speed ratio between baseline phase and end point phase. One RPM feature (maximum daily step in recovery phase) is skipped because it is collinear and weaker than the SD of recovery phase daily step counts. For the nomogram model we used the following features: clinical N stage, pathological T stage, and surgical margin clear. Clinical N stage was N1 for all patients as this was the inclusion criteria for the study; hence, this feature was not relevant in the AI models. This is all summarized in Table 4.

**Table 4.** Final features to use for the models. CP indicates model that uses CP features, CP+RPM indicates model that uses CP and RPM features as defined in the previous paragraph, and nomogram indicates standard nomogram-based model that uses CP features.

Features	Phase of collection		
	Baseline	Recovery	End point
RPM^a^			
Daily step count over a monitoring period			
SD	Not used	CP^b^+RPM	CP+RPM
Chair-to-stand test			
Number of times	Not used	Not used	CP+RPM
Recovery speed			
Chair-to-stand test—End point to baseline	Not used	Not used	CP+RPM
QLQ-C30^c^			
Constipation	CP+RPM	Not used	Not used
QLQ-BLM30^d^			
Urinary problems	Not used	CP+RPM	CP+RPM
Abdominal problems	CP+RPM	Not used	Not used
CP			
Demographics			
Age	CP, CP+ RPM	Not used	Not used
BMI	CP, CP+RPM	Not used	Not used
Gender	CP, CP+RPM	Not used	Not used
ECOG^e^	CP	Not used	Not used
Clinical			
N stage	Nomogram	Not used	Not used
Cancer type—urothelial carcinoma	CP	Not used	Not used
Cancer type—squamous carcinoma	CP	Not used	Not used
Surgical			
Margin clear	CP, nomogram	Not used	Not used
Number of positive resected nodes	CP, CP+RPM	Not used	Not used
Pathological			
T stage	CP, CP+RPM, nomogram	Not used	Not used
Flat carcinoma in situ	CP	Not used	Not used
Inpatient			
Number of theater visits	CP	Not used	Not used
Length of stay	CP	Not used	Not used
Days taken to return to regular diet	CP	Not used	Not used

a RPM: remote patient monitoring.

b CP: clinical-pathological.

c QLQ-C30: European Organisation for Research and Treatment of Cancer QLQ-C30.

d QLQ-BLM30: European Organisation for Research and Treatment of Cancer QLQ-BLM30.

e ECOG: Eastern Cooperative Oncology Group performance status.

### Model Performance and Explanation

**Figure 4.**
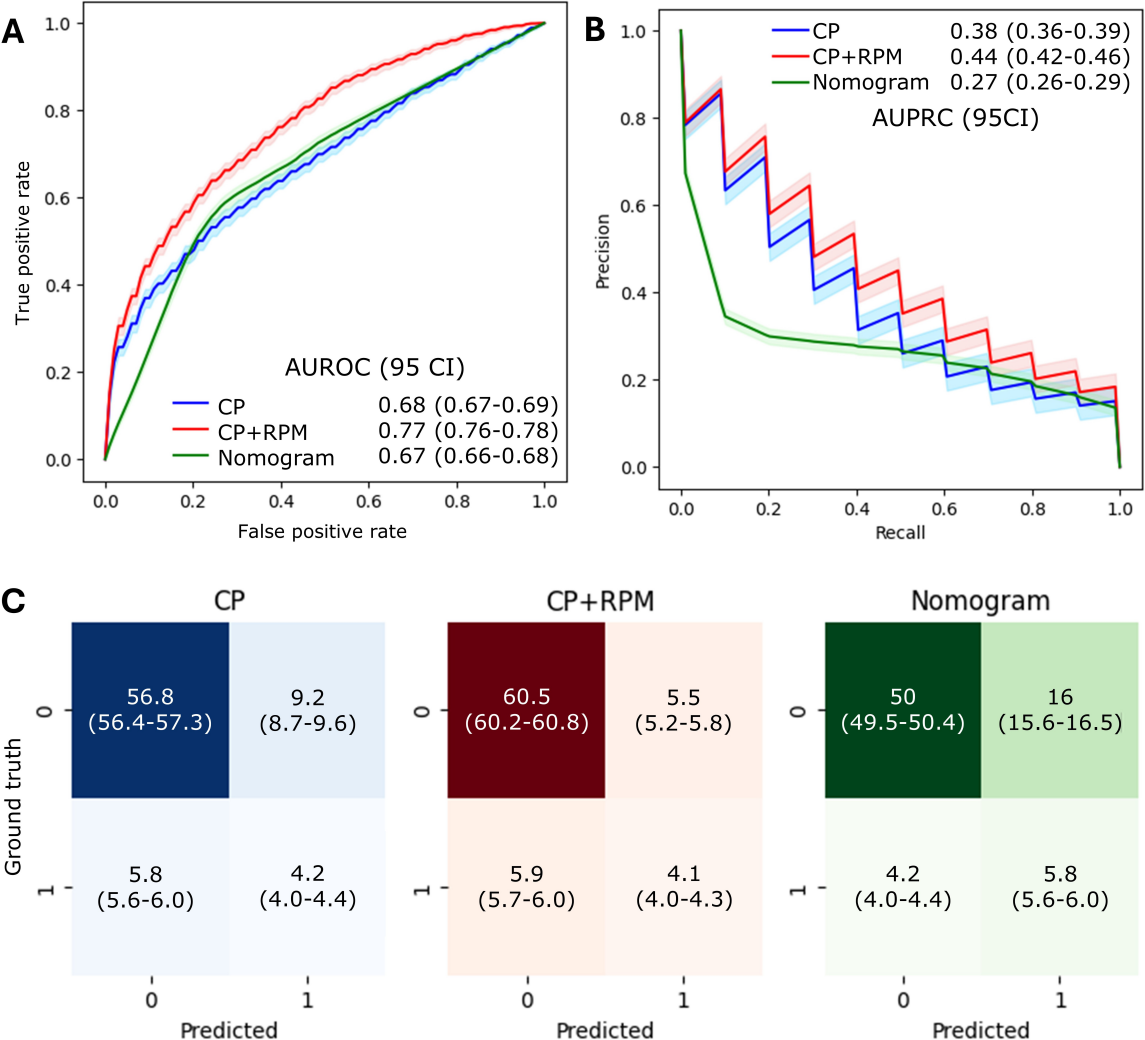
Prediction performance of the 3 models on the testing set. (**A**) Receiver operating characteristic curves (ROCs). Shaded area: 95% CI of ROCs; (**B**) precision-recall curves; and (**C**) confusion matrix. Values within parentheses show 95% CIs. AUPRC: area under the precision-recall curve; AUROC: area under the receiver operating characteristic curve; CP: clinical pathological model; CP+RPM: clinical pathological+ remote patient monitoring model; nomogram: clinical nomogram-based model.

Figure 4A shows the prediction of the developed 3 models—CP, CP+RPM, and nomogram models—in terms of ROC curves. On average, across 200 bootstrap runs, the nomogram and CP models achieved average AUROC scores of 68% and 67%, respectively, while the CP+RPM model scored the highest AUROC score at 77%. When compared with the CP model, the CP+RPM model demonstrates a 9% absolute (13% relative) increase in AUROC. Compared with the nomogram, the CP+RPM model showed a 10% absolute (15% relative) increase in AUROC. The 95% CIs for the AUROCs are reported in the figure legend and are plotted on the figure, demonstrating that the 95% CI for the CP+RPM model has a clear separation from the other 2 models, indicating a statistically significant improvement.

Figure 4B illustrates the PRC for the 3 models. The CP+RPM model was the best-performing, achieving an AUPRC of 44%. Compared with the CP model, which achieved 38% AUPRC, the CP+RPM model has a 6% absolute (16% relative) increase. Compared against the nomogram method, the CP+RPM model has a 17% absolute (63% relative) increase. The 95% CIs for the 3 models have no overlap in the center area, indicating statistically significant improvement in common scenarios. PRC curves for the CP and CP+RPM have a zigzag shape due to the imbalance in the dataset which has relatively few positive samples (13%).

Figure 4C displays the confusion matrices for the CP+RPM, CP, and nomogram models, which are evaluating all 3 models in predicting death (positive, >0.5) and survival (negative, <0.5). We see from the figure that while the nomogram is slightly better at predicting positive rates, the CP+RPM model is overall the best and demonstrates a significantly better true negative rate than both CP and nomogram models.

**Figure 5.**
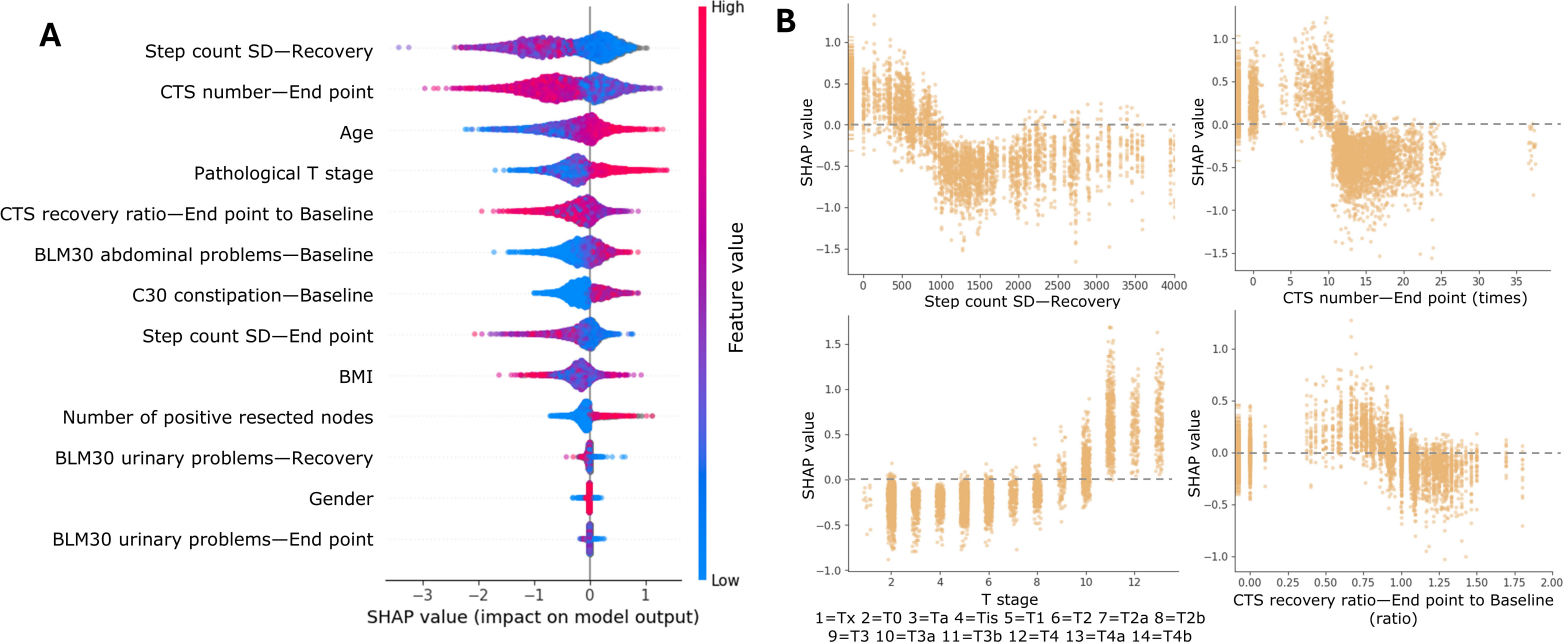
Explanation of the clinical-pathological + remote patient monitoring (CP+RPM) model on the testing set. (**A**) Summary of the feature explanation of the CP+RPM model. This plot illustrates the influence of each feature on the likelihood of mortality as predicted by the model. (**B**) Examples of the relationship between SHAP values and feature values. This subplot demonstrates how specific feature values correlate with SHAP values, illustrating how changes in feature values can impact the predicted risk of mortality. BLM30: European Organisation for Research and Treatment of Cancer QLQ-BLM30; C30: European Organisation for Research and Treatment of Cancer QLQ-C30; CTS: chair-to-stand test; SHAP: Shapley Additive Explanations.

Figure 5A is a SHAP summary plot of the CP+RPM model on the test set. This plot illustrates the influence of each feature on the likelihood of mortality as predicted by the model. Each point in the plot represents the SHAP value of a particular feature for an individual patient, indicating how much that feature influenced the predicted outcome. When multiple points share the same × value, they are stacked to reflect density. Features with positive SHAP values increase the predicted risk of mortality, while negative SHAP values decrease it. Features with long tails indicate strong importance in the model’s predictions. The plot illustrates that activity- and mobility-related features (eg, step count SD and CTS test results) have the highest importance. Survey responses also rank high in importance, which aligns with the feature correlation analysis shown in Figure 3.

Figure 5B explores the correlation between SHAP values and 4 key features: step count variance in the recovery phase, QLQ-C30 constipation score at baseline, CTS number at the end point phase, and CTS recovery ratio at the end point phase relative to baseline. The top left subfigure indicates that very low step count variance (less than 0.1e6 step^2^) at the recovery phase (5 weeks after RC) is associated with a higher risk of mortality. The top right subfigure indicates that patients with constipation problems at the baseline phase have a higher risk of mortality. The bottom left subfigure indicates that those who cannot perform more than 10 CTS repetitions at the end point phase (12 weeks after RC) have a higher risk of mortality. The bottom right subfigure indicates that those with poorer recovery from RC (recovery ratio <0.8 in comparison with baseline) tend to have a higher risk of mortality.

### Use Case Analysis

**Figure 6.**
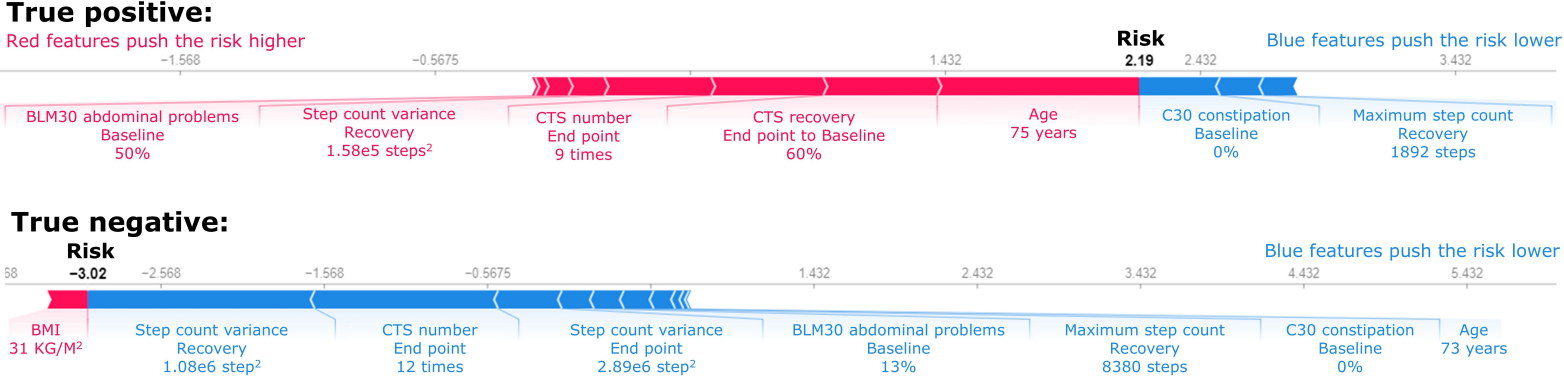
Case analysis of 2 independent predictions. Blue features push the risk lower and red features push the risk higher. BLM30: European Organisation for Research and Treatment of Cancer QLQ-BLM30; CTS: chair-to-stand test; C30: European Organisation for Research and Treatment of Cancer QLQ-C30.

Figure 6 shows 2 prediction examples, illustrating a positive and a negative case. In the positive case, the patient passed away 7 months after the initial surgery due to urosepsis and bladder cancer. Despite having no baseline constipation issues (C30 constipation subdomain=0) and achieving a relatively high daily maximum step count (1892 steps) during the recovery phase, these factors were not sufficient to offset the elevated risk driven by other factors. The patient’s advanced age (75 years), slow recovery in the CTS test (60% of the baseline level), and below-average mobility (end point CTS=9 repetitions) contributed predominantly to the high predicted mortality risk. Risk of 2.19 corresponds to 89.9% predicted probability that the patient will die.

In the negative case, the patient exhibited multiple factors contributing to a lower predicted risk. This patient demonstrated high mobility, with a recovery step count SD of 1040 steps, an end point CTS of 12 repetitions, and an end point step count SD of 1701 steps. The patient’s recovery phase daily maximum step count was 8380 steps. Additionally, only minor issues were observed at the baseline, such as a baseline BLM30 abdominal problem score of 13% and no baseline constipation issues (C30 constipation subdomain=0%). These factors collectively contributed to the lower mortality risk. Risk of −3.02 corresponds to 4.7% predicted probability that the patient will die.

## Discussion

### Principal Findings

This study provides an exploratory analysis of the value of AI RPM in predicting long-term survival following RC for bladder cancer. By incorporating dynamic, real-time data from patients’ daily activity and functional recovery, our AI RPM model demonstrated significant improvements over traditional CP models. Some of the key RPM predictive features—such as daily step count variance and CTS test results—likely capture ongoing recovery processes that static clinical metrics alone cannot.

The activity level of RPM features observed in this study follows a particular pattern of recovery over the 12 weeks postsurgery: baseline level—low level—medium level—baseline level. This is consistent with findings from previous studies; for example, pre- and postoperative daily step counts collected in this experiment follow similar distributions as described by van der Meij et al [21]. Initially, activity levels decreased due to surgical procedures, but daily steps gradually returned to preoperative levels over several weeks. This expected pattern of recovery was followed by all RPM features (activity and self-reported quality-of-life surveys), and it is in the deviation from this pattern that the differences between the survival and the nonsurvival groups were found both through correlation analysis and via the AI model and the SHAP analysis of the feature importance.

In particular, we discovered that RPM features have much higher correlation coefficients than CP features. While certain CP features such as age, pathological T stage, and the number of resected positive nodes were significant, the majority of RPM features frequently exhibited much higher correlation coefficients with mortality risk, in particular, QLQ-BLM30 urinary problems, the number of CTS repetitions, step count variance, maximum daily step count, BLM30 abdominal problems, and CTS recovery speed. Specifically, 36 RPM features had correlation coefficients exceeding 0.1 (implying at the minimum a measurable, although weak, relationship between the feature and the survival) compared with only 10 CP features.

The surgery type (robotic vs open RC) did not show statistically significant difference across 2 groups (Table 1; *P*=.80). The correlation coefficient of surgery type was not significant enough to be included in either CP or CP+RPM models. Although surgery type is prognostically important for survival length as shown by Catto et al [17], it was not a significant predictor in diagnostic models aimed at predicting mortality within a fixed time frame.

Most importantly, we found that the inclusion of RPM features resulted in improved survival prediction by 10% in absolute terms (15% relative) as can be seen from the comparison between the CP+RPM and CP models. The CP+RPM model achieved an AUROC of 77%, and also significantly outperformed the nomogram-based prediction model, which scored 67% on the same dataset. Additionally, the CP+RPM model demonstrated a 17% absolute (63% relative) increase in the AUPRC compared with the nomogram model. That said, the nomogram model did quite well compared with both AI models, given its relative simplicity and the use of only 3 features. However, this is to be expected since our 2 AI models were trained on a very small dataset presented here with only 33 positive cases, while the nomogram model was developed on a much larger cohort with 640 patients and 198 positive cases [29]. Once the dataset for the training of the AI models is larger, it is likely that the AI model accuracy will even further improve. In particular, as the size of the dataset increases, it will be possible to include more features in the AI models—at the moment we were limited to 10 to avoid overfitting, overdimensionality, and noise. In the future, it is highly likely that the data and the AI models will be much larger and able to accommodate very personalized and extremely accurate predictions.

SHAP analysis provided key insights into the relative importance of individual features in predicting mortality, highlighting the substantial contribution of mobility-related features captured through RPM. Specifically, features such as step count variance and the number of CTS test repetitions emerged as the most important, ranking higher than age—a commonly important feature in survival prediction [33]. This finding aligns with recent research by Low et al [34] (2026), which demonstrated that activity intervention can substantially improve the quality of life of patients. This is further exemplified in the use case analysis: in the positive case, the patient exhibited a slow recovery, as indicated by fewer CTS repetitions (60% of baseline) and lower mobility (end point CTS=9), which SHAP identified as highly influential in elevating the mortality risk. Conversely, in the negative case, high mobility levels, with 12 CTS repetitions and a significant step count variance, strongly reduced the predicted mortality risk. The interpretability of the model offers significant potential for clinical translation, particularly in the context of shared decision-making. While accurate prediction is clinically useful, the ability to visualize the specific impact of modifiable lifestyle factors empowers both clinicians and patients to act on these predictions [34].

Interestingly, we observed that the compliance rate in wearing the monitoring device during the recovery phase after surgery could be a practically meaningful indicator of survival. The overall compliance rate in our study was 68%, which is comparable with the 65% compliance reported by Low et al [35] for patients with cancer in a similar context (postsurgical, postdischarge, and unsupervised by clinicians). Compliance during this critical recovery period was 73% in the survivor group compared with 57% in the nonsurvivor group, with a *P* value of .07, indicating a potential trend. Although not reaching the conventional statistical significance threshold of *P*=.05, the 16% difference in compliance suggests a possible distinction between the groups that could have practical relevance. Although compliance was not among the top 10 most important features in our model, it still provides an interesting insight into behavioral differences between the 2 groups.

### Limitations

Our study is subject to several limitations. First, the dataset was imbalanced (13% positive cases), which can negatively impact model performance by causing bias toward the majority class. To address this, we applied class-weighting techniques, assigning higher penalties to misclassifications in the minority class (positive) compared with the majority class (negative), thereby encouraging the model to better distinguish between them. Second, the analysis was based on a small dataset of 252 patients. While including more patients, and in particular more positive cases, would increase the credibility of our results, this study represents one of the largest prospective patient cohorts with longitudinal monitoring after RC, which adds to its value despite its size. Third, variability in sensor wearing time may have introduced errors in activity measurement, although we mitigated this by filtering out monitoring phases with insufficient data and aggregating data across phases.

Missingness in RPM data represents another limitation. Missing data arose primarily from not wearing the device and device loss in shipping during the COVID-19 pandemic. Such missingness may introduce bias and reduce statistical power, especially if it is not missing at random. That said, missingness was treated as features in the CP+RPM model and suggested added value to the mortality prediction. Additionally, the baseline models (nomogram and CP-only model) are unaffected by missing data because they rely exclusively on clinical-pathological variables. In the future, prospective validation with predefined data collection protocols and external cohorts will be conducted to confirm model robustness and clinical usefulness prior to real-world deployment.

Ideally, the model would be validated using external data. However, this type of dataset is extremely rare due to the complexity and heterogeneity of the patient population and the clinical burden of data collection. The dataset used in this study was itself collected across multiple centers, providing a degree of institutional and clinical diversity. Nevertheless, the total number of patients remained small, which precluded holding out an independent external cohort without substantially compromising model training and evaluation. In the future, we will include an external validation dataset for this AI RPM model evaluation.

### Future Work and Conclusions

Future work will focus on involving more patient data, external validation, building a rigorous data collection pipeline, investigating the potential bias associated with missing data, improving hyperparameter selection, and validating the result using different data sources. Additionally, future work will investigate whether optimizing wear during clinically and behaviorally informative time windows (eg, the early postoperative recovery phase) can provide sufficient or even superior prognostic information while reducing patient burden. This includes systematic evaluation of patient feedback and acceptability, identification of high-yield monitoring periods, and assessment of how targeted monitoring strategies affect the reliability and interpretability of features derived from remote-sensing data, particularly in vulnerable postsurgical populations.

In summary, our study demonstrates that RPM features significantly enhance long-term survival prediction for patients who have undergone RC. The integration of real-time, dynamic data provided a more accurate and personalized view of patient recovery, improving both the precision and clinical relevance of the model. The SHAP analysis revealed the key importance of dynamic recovery metrics, such as mobility and functional recovery, which were consistently more predictive of long-term survival than static CP features. This in turn provided individualized, transparent explanations for each patient’s predicted risk, which could enhance clinicians’ understanding of which factors most influence postsurgical outcomes. These results underscore the potential for AI-powered RPM tools to create more tailored interventions and improve patient management through enhancing personalized and more efficient patient care before and after cancer surgery.

Finally, the promising results from this study suggest that AI RPM could be effectively applied to other types of cancer surgeries and major surgeries in general. By capturing dynamic aspects of recovery that traditional models cannot, AI-powered RPM holds great potential to revolutionize postoperative care across various surgical contexts, providing clinicians with a powerful tool to improve outcomes and optimize patient recovery.

## Supplementary material

10.2196/68994 Multimedia Appendix 1 Inclusion and exclusion criteria.

10.2196/68994 Multimedia Appendix 2 Photograph of the wearable device and kit issued to patients.

10.2196/68994 Multimedia Appendix 3 A list of subdomains and corresponding questions.

10.2196/68994 Multimedia Appendix 4 Model hyperparameter configurations.

10.2196/68994 Multimedia Appendix 5 Tables showing extended features and extra distribution measurements.

10.2196/68994 Multimedia Appendix 6 Feature correlation heatmap of all feature combinations.

## Abbreviations

AI: artificial intelligence
AUPRC: Area under the precision-recall curve
AUROC: area under the receiver operating characteristic curve
CP: clinical-pathological
CTS: chair-to-stand
ECOG: Eastern Cooperative Oncology Group performance score
EORTC: European Organisation for Research and Treatment of Cancer
GBM: gradient boosting machine
iROC: intracorporeal robot-assisted radical cystectomy vs open radical cystectomy
PRC: precision-recall curve
QLQ-BLM30: EORTC QLQ-BLM30
QLQ-C30: EORTC QLQ-C30
RC: radical cystectomy
ROC: receiver operating characteristic curve
RPM: remote patient monitoring
SHAP: Shapley Additive Explanations
