# Supplementary material for: Artificial Intelligence Remote Patient Monitoring for Predicting Overall Survival for Patients Undergoing Radical Cystectomy for Bladder Cancer: Exploratory Analysis of the Prospective Trial
Source: JMIR AI. 2026 May 20;5:e68994. doi: 10.2196/68994 (PMC13189257; doi:10.2196/68994)
Supplement: Multimedia Appendix 1 [file ai-v5-e68994-s001.docx]

Inclusion criteria:

1. Participants must be over 18 years of age
2. Histopathological confirmation of BC (Urothelial Cell Carcinoma (UCC), Squamous Cell Carcinoma (SCC), adenocarcinoma or rare variant).
3. Carcinoma in situ (CIS) or stage pTa or pT1 or ≥pT2 or mobile bladder mass on bimanual examination under anaesthesia.
4. Node status ≤N1 on imaging criteria or positron emission tomography (PET)–ve outside pelvis.
5. Eastern Cooperative Oncology Group (ECOG) grade 1, 2 or 3.
6. Able to give informed written consent to participate.

Exclusion criteria:

1. Unwilling to undergo cystectomy.
2. Previous abdominal surgery rendered them unsuitable for either iRARC or ORC.
3. Patients with upper urinary tract disease.
4. Concomitant disease that would render the patient unsuitable for the trial.
5. Pregnant or lactating females.
6. Previous radiotherapy for BC.
