# Supplementary figures and images for: Artificial Intelligence Remote Patient Monitoring for Predicting Overall Survival for Patients Undergoing Radical Cystectomy for Bladder Cancer: Exploratory Analysis of the Prospective Trial
Source: JMIR AI. 2026 May 20;5:e68994. doi: 10.2196/68994 (PMC13189257; doi:10.2196/68994)

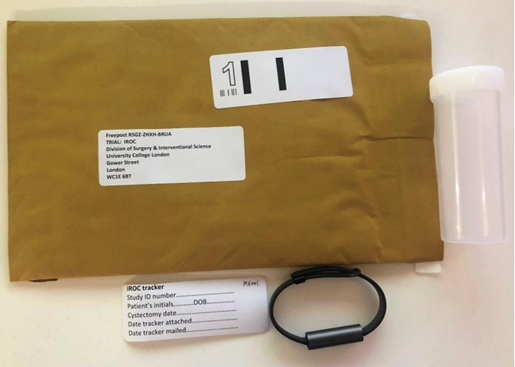

Supplement: Multimedia Appendix 2 [file ai-v5-e68994-s002.png]

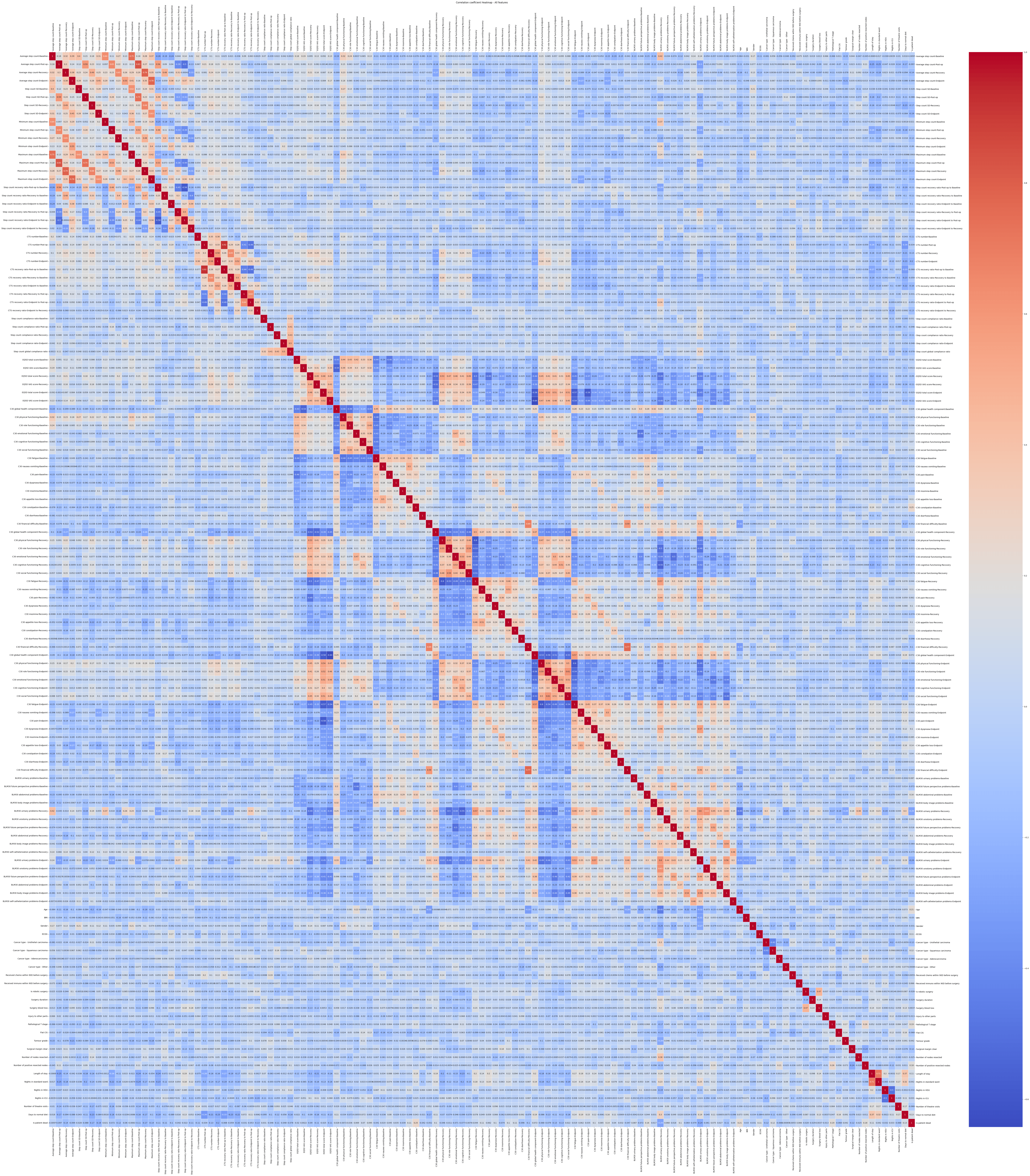

Supplement: Multimedia Appendix 6 [file ai-v5-e68994-s006.png]
