# Supplementary material for: Artificial Intelligence Remote Patient Monitoring for Predicting Overall Survival for Patients Undergoing Radical Cystectomy for Bladder Cancer: Exploratory Analysis of the Prospective Trial
Source: JMIR AI. 2026 May 20;5:e68994. doi: 10.2196/68994 (PMC13189257; doi:10.2196/68994)
Supplement: Multimedia Appendix 3 [file ai-v5-e68994-s003.docx]

| Survey name | Subdomain name | Question no. |
| --- | --- | --- |
| EQ-5D-5L | Quality of Life | 1-5 |
| EQ-5D-5L | EuroQoL visual analogue scale | 6 |
| QLQ-C30 | Global health | 29,30 |
| QLQ-C30 | Physical functioning | 1-5 |
| QLQ-C30 | Role functioning | 6,7 |
| QLQ-C30 | Emotional functioning | 21-24 |
| QLQ-C30 | Cognitive functioning | 20,25 |
| QLQ-C30 | Social functioning | 26, 27 |
| QLQ-C30 | Fatigue | 10,12,18 |
| QLQ-C30 | Nausea and vomiting | 14,15 |
| QLQ-C30 | Pain | 9,19 |
| QLQ-C30 | Dyspnoea | 8 |
| QLQ-C30 | Insomnia | 11 |
| QLQ-C30 | Appetite loss | 13 |
| QLQ-C30 | Constipation | 17 |
| QLQ-C30 | Financial difficulty | 28 |
| QLQ-BLM30 | Urinary problem | 31-37 |
| QLQ-BLM30 | Urostomy problems | 38-43 |
| QLQ-BLM30 | Future perspective | 45-47 |
| QLQ-BLM30 | Abdominal problems | 48,49 |
| QLQ-BLM30 | Body image | 50-52 |
| QLQ-BLM30 | Catheter use problems | 44 |
