# Supplementary material for: Artificial Intelligence Remote Patient Monitoring for Predicting Overall Survival for Patients Undergoing Radical Cystectomy for Bladder Cancer: Exploratory Analysis of the Prospective Trial
Source: JMIR AI. 2026 May 20;5:e68994. doi: 10.2196/68994 (PMC13189257; doi:10.2196/68994)
Supplement: Multimedia Appendix 4 [file ai-v5-e68994-s004.docx]

{

    'max_depth': 4,

    'learning_rate': 0.1,

    'n_estimators': 30,

    'subsample': 0.8,

    'colsample_bytree': 0.9,

}
